# Influence of Anxiety and Depression on Quality of Life of People with Schizophrenia in the Eastern Region of Poland

**DOI:** 10.5402/2012/839324

**Published:** 2012-02-19

**Authors:** Marta Makara-Studzińska, Małgorzata Wołyniak, Karolina Kryś

**Affiliations:** Independent Laboratory of Mental Health, Medical University of Lublin, 20-093 Lublin, Poland

## Abstract

Schizophrenia is the most severe and most debilitating mental illness, which is one of the first ten causes of disability in youth and elderly people. Regarding many consequences that schizophrenia brings for individual and social functioning of ill people, their assessment of the quality of their lives seems to be interesting. The aim of this study is to evaluate the incidence and severity of anxiety and depression as well as analysis of the impact level of anxiety and depression on life quality of people with schizophrenia. A group of patients with schizophrenia from psychiatric centers was involved in a study. A set of methods, included: author's questionnaire, the quality of life scale WHOQOL-BREF, and the hospital anxiety and depression scale (HADS). Anxiety disorders occurred in more than 78% of respondents, while depressive disorders in more than half of respondents. The more severe anxiety and depressive disorders, the lower values were observed in all tested components of quality of life. The study of quality of life of the mentally ill patients should be conducted on a continuous basis in order to explore the current factors influencing the improvement of their psychophysical welfare. It is necessary to promote prohealthy mental lifestyle.

## 1. Introduction

Schizophrenia is a chronic disease characterized by diverse and varied picture of the clinical course. It is undoubtedly the most serious and most debilitating mental illness, which is one of the first ten causes of disability among the young and the elderly. The risk over a lifetime reaches 1% regardless of geographical areas and cultural regions in the world. Usually it begins in adolescence or early adulthood and for most patients is lifelong. In the course of the disease all aspects of human functioning before illness are changing. The incidence is equal in both sexes, but men usually suffer at an earlier age, women in later life. In Poland, schizophrenia affects about 400 thousand people. In the province of Lublin in 2008, the number of patients with schizophrenia was 6674, and the number of newly registered cases of the disease was equal to 1225 [1].

The fact of occurrence of chronic illness and the phenomenon of stigmatisation that is associated with the attitude of the society toward the mentally ill people often makes it impossible for them to start their own family. Among those remaining in the formal marriages or informal relationships the occurrence of the disease usually worsens the relationship with their spouse or partner, with children and other family members.

Their professional life is significantly distorted, their professional status is lowered. The patient is unable to perform their previous roles, their system of values is changed, and needs and life plans are also different. The patients start to break contact with other people, isolation, loss of interest, and emotional ties occur. The difficulties of patients are not limited to episodes of illness, but also concern the period between them.

In accordance with many consequences that schizophrenia brings for individual and social functioning of ill people, their assessment of quality of their lives seems to be interesting.

Systematic researches on the quality of life of patients began only in the eighties. Quality of life in medicine is important when developing new diagnostic and therapeutic methods. Researchers are constantly looking for methods more effective and safe in controlling the disease. Often, however, their use is associated with deterioration in quality of life of patients, it becomes a reason for giving up the treatment, which in turn is equal to its ineffectiveness. Initially, this type of analysis was carried out mainly in the field of oncology. Then researchers drew attention to the concept of quality of life also in other fields of medicine. Patients with chronic diseases like diabetes [2] or epilepsy [3] were tested.

In the case of the mentally ill patients, there were doubts whether the lack of access, presence of psychotic symptoms can affect the patient's assessment of their quality of life and whether that assessment is reliable [4].

Despite all the doubts about the credibility and reliability of assessment of the quality of life conducted by patients themselves, for several years till now the subjective assessment of a patients with schizophrenia is taken increasingly into account and is considered to be the most important. It is fair to say that the assessment of quality of life of patients is one of the most important components of modern medical diagnosis and prognosis and allows for more targeted therapeutic intervention, expressing subjective approach to the patient, in order to meet their real needs.

The main objective of the study was to assess the prevalence and severity of anxiety and depression as well as the impact analysis of level of anxiety and depression on quality of life of people with schizophrenia.

## 2. Material and Methods

Before we started the implementation of the present study, this research project was reported to the Bioethical Committee of the Medical University in Lublin. After receiving a positive review for the presented project of the clinical trial with the no. KE-0254/142/2008, we began to collect the research material.

In the study conducted from July 2009 to April 2010 the group of patients with schizophrenia from community psychiatric centres in Lublin province (Environmental Self-Help Homes, Charitable Society for Aid to the Sick “Misericordia" and the Care and Educational Facility “Misericordia", Daily department of the Neuropsychiatric Hospital in Lublin, Department of Psychiatric Rehabilitation at the Neuropsychiatric Hospital in Lublin and Occupational Therapy Workshops) was involved.

The criteria for inclusion of people with schizophrenia to the study were the following: written consent to participate in the study, diagnosis of schizophrenia according to ICD-10, age from 18 to 65 years, at least one hospitalization in the closed ward, no evidence of CNS damage, the lack of addictions, and contact with the family.

Among 180 distributed questionnaires, 122 were received. Manoeuvrability was 67.8%. 7 questionnaires were improperly filled by people with schizophrenia, and therefore they were excluded from the study. We analyzed 115 correctly completed questionnaires, consisting of a SET OF METHODS for patients with schizophrenia.

A set of methods for people with schizophrenia consisted of five scales:

- author's questionnaire,
- the quality of life scale, a standardized WHOQOL-BREF instrument,
- anxiety and depression scale, a standardized HADS instrument (hospital anxiety and depression scale),
- author's questionnaire was developed for the patient based on knowledge and experience of the author and the available literature.

WHOQOL-Bref scale is a research tool designed for the cognitive and clinical purposes. This scale is an international instrument to assess subjective quality of life. Bref version (the short one) WHOQOL was constructed on the basis of WHOQOL-100. The scale model is based on concepts developed by the WHO quality of life. It is used to measure satisfaction with life of people in the general population and in people suffering from various diseases. WHOQOL-Bref consists of 26 questions and allows to obtain the profile of the quality of life in four domains: physical, psychological, social relationships, and environmental.

The score reflects the areas of individual perceptions of quality of life in these areas. All questions are rated using a 5-point Likert scale. It is the scale of satisfactory reliability and validity, and its usefulness in studies of people with mental disorders has been documented by many works, both in Poland and in the world [5].

Anxiety and depression scale, hospital anxiety and depression scale (HADS), was constructed in 1983 by RP Zigmonda and Snaith [6]. It consists of two separate subscales, one evaluates anxiety (HADS-A) and the second one depression (HADS-D). Each of the subscales contains 7 statements about the current state of the test person. Both subscales are counted and interpreted separately. For each question there are 4 variants of answers; each variant is indicated by a different numerical value (from 0 to 3). Counting is a simple summation of all values for the marked answers, separately for questions about anxiety, and separately for questions about depression. The anxiety scale measures the general anxiety, unfocused on a particular situation, and describes the mood of fear, anxiety, and lack of relaxation.

## 3. Results and Discussion

**Figure 1:**
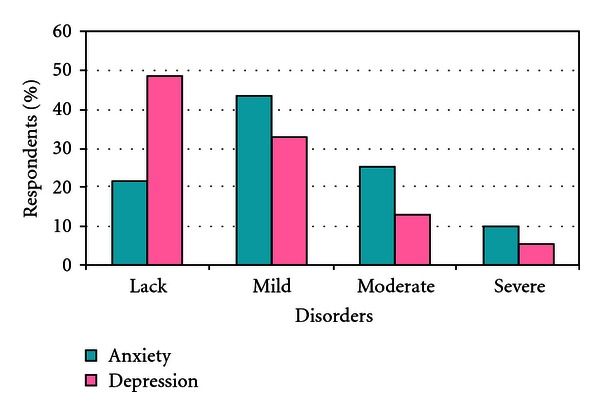
Anxiety and depressive disorders in tested patients with schizophrenia.

In the study group anxiety and depressive disorders were analyzed using the HADS scale. The average level of anxiety was 9.53 (SD = 3.63) and depression 7.58 (SD = 4.09) (Figure 1).

Anxiety disorders were not observed in almost one in five people (21.74%). Mild anxiety disorders occurred in every second (43.48%), moderate in every fourth (25.22%), and severe in every tenth (9.57%) test person.

Depressive disorders were not observed in almost half (48.70%) of respondents. Mild depressive disorders occurred in every third (33.043%), moderate in every eighth (13.04%) test person, and severe in only six (5.22%) respondents.

A correlation between the quality of life of tested patients with schizophrenia and the occurrence of anxiety and depression disorders in these patients was tested. In the study group we looked for correlations between the results in two general questions and areas of quality of life as well as anxiety and depression disorders.

A strong negative correlation was observed between the severity of both anxiety disorders and depression and the values obtained in the overall perception of quality of life, in the general health situation as well as in all areas of quality of life, which means that the more severe anxiety and depressive disorders, the lower values observed in all tested components of quality of life (Table 1).

It is worth noting that the strongest correlations were observed between the level of anxiety and depressive disorders and psychological field (Table 2). 

Respondents in the case of severe anxiety or depression got the lowest scores in all areas of quality of life, while in those without any depressive disorder were observed the highest scores in all areas (Table 3).

It is assumed that depressive disorders occur in 60%–75% of people with schizophrenia. During the first episode and reactivation of psychosis the frequency rate of depressive disorders ranges from 65% to 80%, and during the remission period from 4% to 20% [7].

 All the studies published so far provide evidence that depression and anxiety are strong predictors of quality of life in schizophrenia [8–18].

As well as the impact of depression on quality of life of people with schizophrenia seems to be a stronger predictor than psychotic symptoms. Huppert et al. found that anxiety and depression measured by the scale of BPRS (brief psychiatric rating scale), independently of each other and of other symptoms of schizophrenia, correlate mostly with the subjective quality of life [19].

Similar conclusions are reached by Bechdolf et al. in 2003 in their study on a group of 66 people with schizophrenia; in their case depression most strongly correlated with subjective quality of life of patients [20].

The work of Górna et al. demonstrated that people with schizophrenia with accompanying depression received lower scores in two questions, both general and in all areas of quality of life measured by WHOQOL-Bref scale compared to those without depression. The largest disparity occurred in the field of psychology [21].

In the the work in China from 2008 the level of depression measured using the Hamilton Depression Rating Scale strongly negatively correlated with all domains of quality of life scale WHOQOL-Bref, anxiety measured using BPRS scale only with the field of psychology [22]. Similar results were obtained by the author in his earlier work from 2007. Anxiety and depression strongly correlated with overall quality of life and with all areas of WHOQOL-Bref scale.

Researchers from Portugal in 2008 came to similar conclusions: the level of depression as measured by the Hamilton Depression Rating Scale strongly negatively correlated with the field of psychological and physical scale WHOQOL-Bref [23].

Additionally, it also appears that those patients with concomitant depression are more aware of the consequences of their disease and treatment efficacy. Depressive symptoms can occur as a consequence of the realization of loss, humiliation, shame, and an expression of blame because of their mental state.

Similar conclusions are presented in this work. Correlations between the severity of anxiety disorders and depression and the values obtained in the overall perception of quality of life, in the general health condition as well as in all areas of quality of life, were pointed out which means that the more severe anxiety and depressive disorders, the lower values were observed in all tested components of quality of life. It is worth noting that the strongest correlations were observed between the level of anxiety and depressive disorders and psychological field including elements of the subscales: appearance, negative feelings, positive feelings, self-esteem, spirituality/religion/personal faith, and thinking/learning/memory/concentration.

## 4. Conclusions

The study of quality of life of the mentally ill people should be conducted on a continuous basis in order to explore the current factors influencing the improvement of their mental and physical welfare. This should be valuable information for managers of medical institutions and medical personnel.

Moderate and severe anxiety disorders occurred among 34.79% of people with schizophrenia, while moderate and severe depressive disorders in 18.26% of people with schizophrenia. These individuals require consultation with a psychologist or psychiatrist and consideration of implementation of appropriate pharmacological or psychotherapeutic interactions. Efforts should be made to compensate these disorders, because the more severe anxiety and depressive disorders, the worse the values observed in all areas of quality of life.

It is necessary to promote prohealthy mental lifestyle and encourage participation in the preventive activities in mental illness, particularly in the eastern region of Poland.

The quality of life of people with schizophrenia and their families is one of the major subjects that are currently being considered in both theory and practice. The need to ensure the quality of life results from the fact that life and human health are the highest value.

## Figures and Tables

**Table 1:** The overall perception of quality of life of patients with schizophrenia, depending on the occurrence of anxiety and depressive disorders.

HADS		WHOQOL-BREF—overall quality of life		Statistical analysis	
Anxiety	Bad	Either good nor bad	Good		
Standard (%)	1 (6,25%)	3 (5,36%)	21 (48,84%)		
Mild disorders (%)	4 (25,00%)	30 (53,57%)	16 (37,21%)		
Moderate disorders (%)	8 (50,00%)	17 (39,36%)	4 (9,30%)	*P* < 0,001***	*χ* ^2^ = 37,54
Severe disorders (%)	3(18,75%)	6(10,71%)	2(4,65%)		

HADS Depression	Bad	Neither good nor bad	Good	Statistical analysis	

Standard (%)	3 (18,75%)	20 (35,71%)	33 (76,74%)		
Mild disorders (%)	5 (31,25%)	24 (42,86%)	9 (20,93%)		
Moderate disorders (%)	4 (25,00%)	10 (917,86%)	1 (2,33)	*P* < 0,001***	**χ*^2^* = 36,46
Severe disorders (%)	4 (25,00%)	2 (3,57%)	0 (0,00%)		

**Table 2:** The overall perception of the own health in patients with schizophrenia, depending on the occurrence of anxiety and depressive disorders.

HADS			WHOQOL-BREF—satisfaction with the health condition		Statistical analysis	
Anxiety	Very dissatisfied	Dissatisfied	Neither satisfied nor dissatisfied	Satisfied		
Standard (%)	0 (0,00%)	5 (14,29%)	8 (17,02%)	12 (42,86%)		
Mild disorders (%)	1 (20,00%)	15 (42,86%)	23 (48,94%)	11 (39,29%)		
Moderate disorders (%)	2 (40,00%)	10 (28,57%)	13 (27,66%)	4 (14,29%)	*P* < 0,05*	**χ*^2^* = 18,51
Severe disorders (%)	2 (40,00%)	(14,29%)	3(6,38%)	1(3,57%)		

Depression	Very dissatisfied	Dissatisfied	Neither satisfied nor dissatisfied	Satisfied	Statistical analysis	

Standard (%)	2(40,00%)	12(34,29%)	21(44,68%)	21 (75,00%)		
Mild disorders (%)	0 (0,00%)	14 (40,00%)	20 (42,55%)	4 (14,29%)		
Moderate disorders (%)	2 (40,00%)	6 (17,14%)	5 (10,64%)	2 (7,14%)	*P* < 0,05*	**χ*^2^* = 20,18
Severe disorders (%)	1 (20,00%)	3 (8,57%)	1 (2,13%)	1 (3,57%)		

**Table 3:** Particular areas of quality of life of patients with schizophrenia, depending on the prevalence of anxiety and depressive disorders.

HADS			WHOQOL-BREF—dimensions		
Lęk		Physical dimension	Psychological dimension	Social dimension	Environmental dimension
	Śr	14,49	13,33	13,81	14,14
Standard	SD	1,99	2,09	2,98	1,78
	Me	14,29	14,00	13,33	14,00

	Śr	13,51	11,65	12,53	13.05
Mild disorders	SD	1,89	1,86	2,67	1,95
	Me	13,71	11,33	12,00	11,50

	Śr	12,71	10,11	10,67	11,72
Moderate disorders	SD	1,76	2,22	3,00	1,94
	Me	12,57	10,00	10,67	11,50

	Śr	10,34	9,03	9,21	10,36
Severe disorders	SD	1,85	1,72	2,95	2,23
	Me	10,86	9,33	8,00	11,00

Statistical analysis		*P* < 0,001***	*P* < 0,001***	*P* < 0,001***	*P* < 0,001***
		*H* = 26,34	*H* = 35,76	*H* = 21,72	*H* = 28,92

Depression		Physical dimension	Psychological dimension	Social dimension	Environmental dimension

	Śr	14,31	12,77	13,60	13,71
Standard	SD	1,88	2,01	2,86	2,02
	Me	14,29	12,67	13,33	14,00

	Śr	12,83	10,70	11,33	12,12
Mild disorders	SD	1,56	1,49	2,29	1,71
	Me	13,14	10,67	10,67	12,00

	Śr	11,54	9,38	9,33	11,63
Moderate disorders	SD	1,89	2,25	2,89	1,94
	Me	11,43	9,33	9,33	11,00

	Śr	9,71	7,67	8,44	9,50
Severe disorders	SD	1,58	1,01	2,48	2,37
	Me	9,14	7,67	8,00	9,50

Statistical analysis		*P* < 0,001***	*P* < 0,001***	*P* < 0,001***	*P* < 0,001***
		*H* = 35,44	*H *= 45,27	*H* = 34,32	*H* = 26,73
